# Presynaptic Molecular Determinants of Quantal Size

**DOI:** 10.3389/fnsyn.2016.00002

**Published:** 2016-02-08

**Authors:** Shigeo Takamori

**Affiliations:** Laboratory of Neural Membrane Biology, Graduate School of Brain Science, Doshisha UniversityKyoto, Japan

**Keywords:** synaptic vesicle, quantal size, VGLUT, VGAT, V-ATPase

## Abstract

The quantal hypothesis for the release of neurotransmitters at the chemical synapse has gained wide acceptance since it was first worked out at the motor endplate in frog skeletal muscle in the 1950’s. Considering the morphological identification of synaptic vesicles (SVs) at the nerve terminals that appeared to be homogeneous in size, the hypothesis proposed that signal transduction at synapses is mediated by the release of neurotransmitters packed in SVs that are individually uniform in size; the amount of transmitter in a synaptic vesicle is called a quantum. Although quantal size—the amplitude of the postsynaptic response elicited by the release of neurotransmitters from a single vesicle—clearly depends on the number and sensitivity of the postsynaptic receptors, accumulating evidence has also indicated that the amount of neurotransmitters stored in SVs can be altered by various presynaptic factors. Here, I provide an overview of the concepts and underlying presynaptic molecular underpinnings that may regulate quantal size.

## Introduction

Synaptic transmission requires the release of neurotransmitters from presynaptic terminals. Since the pioneering work by Katz and colleagues in the frog neuromuscular junction and the morphological identification of synaptic vesicles (SVs) of apparently homogeneous size at presynaptic terminals by electron microscopy, neurotransmitters have been believed to be packed in SVs and released from them as discrete and uniform “quanta”. However, emerging evidence has suggested that quantal response, which is a postsynaptic current elicited by the fusion of a single vesicle, in the mammalian central nervous system (CNS) exhibits a certain degree of variation. Because postsynaptic receptors at mammalian glutamatergic synapses are not usually saturated by release of neurotransmitters from a single SV, possible sources of the variations in quanta are presynaptic. In fact, detailed investigations at central glutamatergic synapses called the calyx of Held have revealed that the vesicular glutamate concentration is a plausible source of quantal variations (Wu et al., 2007). In this review, I will introduce our current molecular knowledge and the possible presynaptic determinants responsible for the regulation of quantal size.

## Minimal Molecular Complexes for the formation of Quantum in SVs

**Figure 1:**
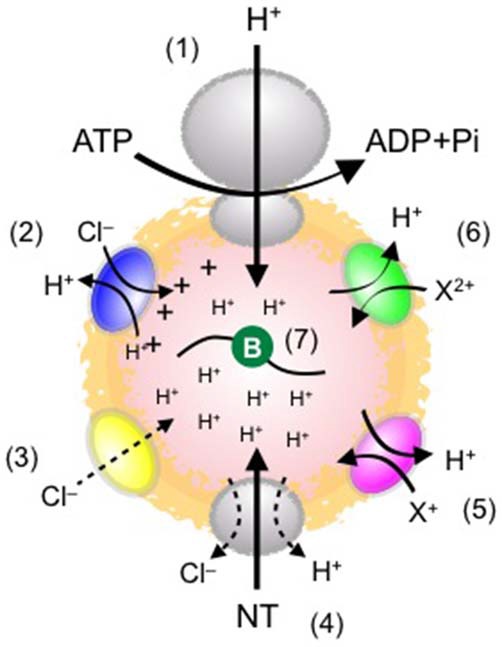
**Schematic diagram of possible elements influencing ΔμH^+^ and quantal size.** (1) Vacuolar-type H^+^ ATPase (V-ATPase), which drives neurotransmitter transport into SVs. ATP concentrations affect the proton-transporting activity. (2) ClC-type Cl^−^ channel family. Some ClC isoforms operate as a 2Cl^−^/H^+^ exchanger. This stoichiometry seems to alkalize the vesicle lumen, but may also dissipate ΔΨ, thereby facilitating ΔpH. (3) A putative Cl^−^ channel. This provides a shunting current for H^+^, leading to acidification. The molecular identity of the Cl^−^ conductance has yet to be determined. (4) Vesicular neurotransmitter transporters. Their expression levels affect quantal size. How transporters are activated by H^+^ and how Cl^−^ modulates the transport remain obscure. (5) Cation/H^+^ exchanger. This activity potentially dissipates ΔpH and facilitates ΔΨ, leading to an increase in ΔΨ-driven glutamate transport. (6) Divalent cation/H^+^ exchanger. This activity plays essentially the same role as (5). The molecular identity and physiological relevance of the system remain unknown. (7) Luminal buffers (symbolized by green circle). The buffering capacity of the lumen conferred by intrinsic proteins, lipids, and the intravesicular solution affects the formation of ΔpH (presumably also ΔΨ), thereby influencing quantal size.

Neurotransmitters are usually synthesized in the presynaptic cytoplasm and are concentrated into SVs by the activity of vesicular transporters specific for the respective neurotransmitters. All known vesicular transporters responsible for neurotransmitter uptake into SVs utilize a proton electrochemical gradient (ΔμH^+^) generated by the vacuolar-type H^+^ ATPase (V-ATPase). The V-ATPase consists of at least 13 subunits with a total molecular weight of ~800 kDa and represents the largest molecular complex on SVs. The V-ATPase is divided into two functionally distinct portions. The V_1_ part consists of one large transmembrane protein (a1 subunit) in association with several globular A and B subunits and catalyzes ATP hydrolysis, releasing energy for proton transfer. The V_0_ part forms a ring-like structure in the membrane and provides a proton permeation pathway. By transferring protons into the SV lumen, the V-ATPase generates both a pH gradient (ΔpH) and a membrane potential (inside positive voltage referred to ΔΨ) across the SV membrane. The vesicular transporters for classical neurotransmitters utilize ΔpH, ΔΨ, or both, depending on the neurotransmitter type. Five classes of vesicular transporters have been cloned and molecularly characterized so far, including two vesicular monoamine transporters (VMAT1, VMAT2), vesicular acetylcholine transporter (VAChT), three vesicular glutamate transporters (VGLUT1–3), vesicular GABA/glycine transporter (VGAT; also referred to as vesicular inhibitory amino acid transporter, VIAAT; Edwards, 2007), and vesicular nucleotide transporter (VNUT; Sawada et al., 2008). Biochemical assays using isolated vesicles in the presence of compounds that selectively dissipate either ΔpH or ΔΨ revealed energy requirements for their uptake. The uptake of cationic transmitters such as acetylcholine and biogenic amines largely depends on ΔpH, whereas that of anionic transmitters such as glutamate predominantly depends on ΔΨ. The uptake of zwitterionic and therefore neutral transmitters such as GABA and glycine depends on both ΔpH and ΔΨ. Despite extensive studies on the bioenergetics of the transport process, the details of the mechanisms of transport, especially how H^+^ differentially drives transport of different neurotransmitters, remain enigmatic. In addition to neurotransmitter transporters and the V-ATPase, potential modulators that may alter the driving force for neurotransmitter uptake also play a role, including ion channels/transporters on SVs (Figure 1). In this review, I will introduce key components that underlie the formation and regulation of quanta with a special focus on glutamate and GABA, the major excitatory and inhibitory neurotransmitters in the mammalian CNS, respectively.

## Expression Level of the Transporters

Intuitively, we expect that expression of transporter proteins on a SV will affect the rate of transport rather than the amount of transmitters at equilibrium. However, overexpression of vesicular transporters in primary cultured neurons and *in vivo* in fruit fly embryos as well as other model systems such as PC12 cells results in an increase in neurotransmitter content in individual vesicles (Pothos et al., 2000; Daniels et al., 2004; Wojcik et al., 2004; Wilson et al., 2005; Table 1). In some cases, enlargement of vesicles is associated with an increase in quantal size (Daniels et al., 2004), although it is unknown whether this can be explained simply by swelling of the vesicles due to alterations in osmolality or if insertion of phospholipids into the vesicles is also involved. In contrast to this view, analyses of VGLUT heterozygous mice revealed somewhat conflicting results. Suppression of VGLUT1 in hippocampal neurons derived from VGLUT1^+/−^ mice does not produce a significant reduction in miniature excitatory postsynaptic currents (mEPSC; Fremeau et al., 2004; Wojcik et al., 2004), whereas VGLUT2^+/−^ neurons that originate from the striatum exhibit reduced mEPSCs (Moechars et al., 2006). Nevertheless, because each SV contains ~10 copies of VGLUT, a robust change in expression levels may be necessary to induce detectable changes in quantal size (Takamori et al., 2006).

**Table 1:** **Gene manipulations of vesicular transporters/channels influence quantal size**.

Gene	Manipulation	Preparation	Quantal size	Reference
VGLUT1	Overexpression	Mouse hippocampal culture	Glu ↑	Wilson et al. (2005) and
				Wojcik et al. (2004)
	Heterozygous	Mouse hippocampal culture	Glu →	Wojcik et al. (2004) and
				Fremeau et al. (2004)
	Knockout	Mouse hippocampal culture	Glu ↓	Wojcik et al. (2004)
	Knockout	Mouse hippocampal culture	Glu →	Fremeau et al. (2004)
VGLUT2	Heterozygous	Mouse striatal culture	Glu ↓	Moechars et al. (2006)
	Knockout	Mouse striatal culture	Glu ↓	Moechars et al. (2006)
DVGLUT*	Mutants	Neuromuscular junction	Glu → or zero	Daniels et al. (2006)
	Overexpression^#^	Neuromuscular junction	Glu ↑	Daniels et al. (2004)
VGAT	Heterozygous	Mouse striatal culture	GABA →	Wojcik et al. (2006)
	Knockout	Mouse striatal culture	GABA ↓	Wojcik et al. (2006)
VMAT2	Overexpression^#^	Rat ventral midbrain culture	Dopamine ↑	Pothos et al. (2000)
ClC-3	Knockout	Acute hippocampal slice	Glu →, GABA →	Stobrawa et al. (2001)
	Knockout	Acute hippocampal slice	GABA ↓	Riazanski et al. (2011)
	Knockout^#^	Hippocampal culture	Glu ↑	Guzman et al. (2014)

*DVGLUT stands for drosophila VGLUT. Glu stands for glutamate. #Enlargement of vesicles were observed by electron microscopy.

Is the expression of vesicular neurotransmitter transporters regulated in physiological or pathological conditions? Several lines of evidence have suggested that this is the case. First, *VGLUT1* and *VGLUT2* were originally identified as genes that are up-regulated upon stimulation in neurons and endocrine cell lines (Ni et al., 1994; Aihara et al., 2000). Second, VGLUT1 expression is developmentally up-regulated, which seems to be accompanied by an increase in mEPSCs (De Gois et al., 2005; Yamashita et al., 2009). Third, expression of VGLUT1, VGLUT2, and VGAT in cultured hippocampal neurons is altered by manipulations that change neural activities, i.e., exposure to tetrodotoxin and antagonists for the respective neurotransmitter receptors (De Gois et al., 2005). Fourth, VGLUT3 expression in some brain regions is very transient, resulting in biphasic expression profiles (Gras et al., 2005). Finally, in addition to the total expression levels, the extent of VGLUT sorting to the plasma membrane may regulate the vesicular level of VGLUT proteins, which oscillate during light/dark cycles (Darna et al., 2009). Collectively, these observations suggest that temporal regulation of expression of vesicular transporters for neurotransmitters scales and shapes synaptic transmission and thus network activity in the brain.

## Regulation of the Proton Electrochemical Gradient

### ATP

Although little is known about regulation of V-ATPase activity *in vivo*, several factors affect the activity, and therefore, neurotransmitter uptake. First, cytoplasmic ATP concentrations may be a key factor in regulation. In isolated SVs, glutamate uptake reaches a plateau with 2 mM ATP, which also requires Mg^2+^ in low mM concentrations (Naito and Ueda, 1985). The cytoplasmic ATP concentration in the presynaptic terminals of rat hippocampal cultured neurons is ~2 mM in resting conditions. During neural activity, as cytoplasmic ATP is rapidly consumed mainly by the process of endocytosis, ATP concentrations decrease to ~1 mM during repetitive stimulations (Rangaraju et al., 2014). Therefore, the physiological concentration range of ATP may impact the activity of V-ATPase, which in turn alters the kinetics and/or extent of glutamate refilling of SVs. Furthermore, in some pathological conditions such as anoxia, hypoglycemia, and ischemia, dramatic ATP breakdown may occur, leading to reductions in V-ATPase activity and neurotransmitter refilling (Santos et al., 1996).

### Cl^−^ Channel

V-ATPase is an electrogenic pump, and therefore, the pump does not transport much H^+^ unless counter-ion movements across the SV membrane are present that abolish the voltage due to the H^+^ flux (Moriyama and Nelson, 1987). Studies with biochemical assays using acridine orange as a pH gradient indicator have shown that membrane-permeable Cl^−^ confers a shunting current for the H^+^ movement, which inhibits the formation of ΔΨ, and in turn, promotes the pH gradient (Maycox et al., 1988; Cidon and Sihra, 1989; Tabb et al., 1992). Accordingly, with high extravesicular (cytosolic) Cl^−^, uptake of cationic neurotransmitters is maximal because ΔpH is large, whereas glutamate uptake, which is driven primarily by ΔΨ, is minimal (Hell et al., 1990). Interestingly, glutamate uptake in the absence of Cl^−^, where ΔΨ is maximal, does not show the highest activity (Naito and Ueda, 1985), leading to the hypothesis that Cl^−^ allosterically activates VGLUTs (Hartinger and Jahn, 1993; Wolosker et al., 1996; see also below); however, contributions of ΔpH or low luminal pH have also been suggested (Tabb et al., 1992; Bellocchio et al., 2000; Schenck et al., 2009). Consistent with biochemical assays with isolated SVs in which the highest glutamate transport is observed in the presence of a Cl^−^ concentration in the range of ~10 mM, efficient glutamate refilling of SVs assessed in the calyx of Held synapses requires 5–30 mM cytosolic Cl^−^ (Hori and Takahashi, 2012). GABA uptake also exhibits biphasic Cl^−^ dependance, albeit to a lesser extent (Hell et al., 1990). Although GABAergic SVs also contain Cl^−^ channel activity (Takamori et al., 2000), the contributions of the channel activity to GABA transport have not been clarified.

The molecular identity of the putative Cl^−^ channel on SVs has not been firmly established. One member of the ClC chloride channel family, ClC-3, is localized in SVs, and the SV fraction derived from ClC-3^−/−^ mice shows a reduced Cl^−^-induced acidification, suggesting that ClC-3 is the Cl^−^ channel on SVs (Stobrawa et al., 2001). In addition, however, both glutamate-induced acidification and glutamate transport activity are dramatically reduced in ClC-3-deficient SV fractions (Note that if ClC-3 were the Cl^−^ channel on SVs, the absence of ClC-3 would result in a reduction in ΔpH, leading an increase in glutamate transport). These phenotypes are associated with a reduction in VGLUT1 protein expression, which may be due to the severe neurodegeneration of the ClC-3^−/−^ brain, including total loss of the hippocampus. Contrary to these biochemical experiments, electrochemical assessments of mEPSCs (and also miniature inhibitory postsynaptic currents, mIPSCs) in acute hippocampal slices from ClC-3^−/−^ mice did not show significant changes compared to wild-type preparations. More recently, the contribution of ClC-3 to glutamatergic neurotransmission was challenged by using cultured hippocampal neurons established from postnatal day 1 mice to avoid alterations in neuronal functions due to neurodegeneration (Guzman et al., 2014). In this preparation, both mEPSCs and evoked EPSCs were increased in ClC-3^−/−^ neurons, supporting the proposal that ClC-3 contributes to SV acidification. Loss of ClC-3 produces additional phenotypes including an increased release probability and an enlargement of SVs, suggesting multiple roles for ClC-3 in presynaptic physiology. Furthermore, contrary to the original report by Stobrawa et al. (2001), ClC-3 may also contribute to GABA transport into SVs. mIPSCs recorded from hippocampal CA1 pyramidal neurons from ClC-3^−/−^ mice have lower amplitudes than those from wild-type mice (Riazanski et al., 2011). Further studies will be necessary to clarify these contradictory results (see Table 1).

An alternative candidate for the Cl^−^ channel on glutamatergic SVs is VGLUTs themselves. When SV fractions derived from VGLUT1^−/−^ mice were examined in an acidification assay, a drastic reduction in Cl^−^-induced acidification was observed both in 3- and 8-week-old mice. In contrast, acidification of SVs derived from 3-week-old ClC-3^−/−^ mice is not significantly different from that of SVs derived from wild-type mice, indicating that the acidification deficit observed in adult ClC-3^−/−^ mice is due to the massive neurodegeneration of the VGLUT1-expressing region (Schenck et al., 2009). The Cl^−^-induced acidification can also be functionally reconstituted in proteoliposomes only when recombinant VGLUT1 protein is included (Schenck et al., 2009; Preobraschenski et al., 2014). An unanswered question is whether one of the two proteins or both is responsible for the Cl^−^ permeation pathway in SVs.

### Cation/H^+^ Exchanger

A proteomic study of purified SVs identified a Na^+^/H^+^ exchanger (Grønborg et al., 2010), and addition of a high concentration of Na^+^ as well as K^+^ decreases ΔpH and increases ΔΨ as intravesicular H^+^ is exchanged by K^+^ or Na^+^, providing favorable conditions for glutamate transport (Goh et al., 2011). Furthermore, replacing cytoplasmic K^+^ with a non-permeable cation attenuates the quantal size recorded from the calyx of Held synapses. Also, increasing the presynaptic Na^+^ concentration facilitates the mEPSC amplitude (Huang and Trussell, 2014), indicating physiological significance for the cation/H^+^ exchange in regulating quantal size. In the case of Na^+^, presynaptic hyperpolarization-activated cyclic nucleotide-gated (HCN) channels may play an important role in controlling the presynaptic Na^+^ concentration. Intriguingly, VGLUTs themselves may confer Na^+^/H^+^ exchanger activity when reconstituted in liposomes, although direct evidence for the movement of monovalent cations through VGLUTs is still lacking (Preobraschenski et al., 2014).

### Ca^2+^/H^+^ Antiport

In analogy to the role of monovalent cations in the regulation of ΔμH^+^, the existence of a putative Ca^2+^/H^+^ antiport system in SVs was demonstrated by using isolated SVs (Gonçalves et al., 1998). Addition of Ca^2+^ (~600 μM) causes a slight reduction in acidification as monitored by acridine orange, indicating H^+^ efflux associated with Ca^2+^ influx (Gonçalves et al., 1999). In an independent experiment, ATP-dependent ^45^Ca^2+^ transport into SVs was also demonstrated. Other divalent cations such as Zn^2+^ and Cd^2+^ also reduce acidification, albeit with higher efficiency. Because both Zn^2+^ and Cd^2+^ attenuate ATP-dependent Ca^2+^ uptake in a concentration-dependent manner, a common transporter may share the substrates (Gonçalves et al., 1999). Because glutamate uptake is predominantly driven by ΔΨ, the Ca^2+^/H^+^ antiport was expected to increase glutamate uptake by converting ΔpH to ΔΨ. However, the effect of Ca^2+^ on glutamate uptake depends on external Cl^−^ concentrations, indicating a complex interplay among ΔpH, ΔΨ, and Cl^−^-dependent activation of VGLUTs (Gonçalves et al., 2001). Interestingly, Ca^2+^-induced “de-acidification” is decreased when the Ca^2+^ sensor protein, synaptotagmin 1, is knocked down in PC12 cells (Cordeiro et al., 2013). The precise mechanisms and physiological relevance in SVs are poorly understood.

### Luminal Buffers

Because SVs are recycled at presynaptic terminals, and newly endocytosed SVs may engulf extracellular solution, the ionic composition as well as the buffering strength of the extracellular solution may impact the formation of ΔμH^+^. In cultured hippocampal neurons expressing synaptopHluorin (a luminal pH sensor), application of 100 mM Tris buffer strongly inhibits re-acidification of newly endocytosed SVs (Ertunc et al., 2007), although the effect on the ΔΨ component remains unclear. Even with lower buffer concentrations, i.e., 15 mM, short-term depression is facilitated, indicating that neurotransmitter refilling is suppressed when a solution with a high buffering capacity is incorporated into SVs during recycling. This was clearly observed when evoked IPSCs were recorded from CA1 pyramidal neurons in an acute hippocampal slice preparation in the presence of 50 mM Tris. Thus, the buffering strength of the extracellular solution critically affects ΔpH (and probably also ΔΨ), leading to alteration of quantal size.

Another source of luminal H^+^ buffer may be intrinsic membrane proteins and lipids that cover the luminal surface of SVs. A recent study estimated that the SV lumen in cultured hippocampal neurons confers relatively high buffering capacity (56 mM/pH) that requires ~1200 H^+^ influx into a SV to establish a ΔpH of 1.8 (Egashira et al., 2015). Because neurotransmitter content and the intravesicular solution (which may be equivalent to the extracellular bath solution) should not contribute much to the luminal buffering capacity, the luminal domains of the intrinsic membrane proteins are likely the acceptor for protons, in good agreement with the extremely high protein density in SVs (Takamori et al., 2006). Although activity-dependent or homeostatic alterations in expression levels of vesicle proteins have often been demonstrated (De Gois et al., 2005; Wilson et al., 2005), possible contributions of these changes in protein levels to regulation of the V-ATPase activity and the quantal size have not been considered.

## Vesicular Synergy

Identification of the proton-driven neurotransmitter transporters and the characterization of their localizations in the nervous system have revealed that some of them are co-expressed in the same presynaptic terminals and even on the same vesicles. In particular, VGLUT3 is expressed in subsets of neurons that had not been considered to be glutamatergic neurons, including GABAergic interneurons in the hippocampus, amacrine cells in the retina, and cholinergic neurons in the striatum (Fremeau et al., 2002; Gras et al., 2002). Furthermore, VGLUT2 is also present in a subpopulation of striatum dopaminergic neurons as well as cultured dopaminergic neurons and contributes to co-release of dopamine and glutamate from the same neurons (Dal Bo et al., 2004, 2008).

In addition to enabling co-release of glutamate with other transmitters, evidence is emerging that suggests that the expression of VGLUTs facilitates the transport of other cationic transmitters. For instance, vesicular transport of glutamate results in increased acetylcholine transport in vesicles isolated from the striatum, and this facilitation is diminished in VGLUT3^−/−^ mice (Gras et al., 2008). Moreover, analysis of the hippocampus and prelimbic cortex from VGLUT2^−/−^ mice demonstrated that glutamate transport into VMAT2-carrying SVs stimulates transport of serotonin (Hnasko et al., 2010). Such synergistic effects of glutamate transport on the uptake of other neurotransmitters were named “vesicular synergy” (see El Mestikawy et al., 2011). Vesicular synergy was also shown for GABA uptake in the presence of glutamate in isolated vesicles (Zander et al., 2010), but such “vesicular synergy” in neurons co-releasing glutamate and GABA turned out to be unlikely, at least in some experimental conditions (Case et al., 2014; Zimmermann et al., 2015). Nevertheless, these distinct synergistic effects of glutamate co-transport on refilling of other transmitters may involve VGLUT-dependent acidification of SVs. In the presence of VGLUTs, both glutamate transport and Cl^−^ influx through VGLUT facilitates vesicle acidification (Maycox et al., 1988; Schenck et al., 2009). Transport of cationic transmitters may be more sensitive to acidification than that of GABA, because transport of cationic transmitters is primarily driven by ΔpH. Conversely, transport of cationic transmitters may prevent efficient glutamate transport into the same SVs. Because transport of cationic transmitters involves net efflux of one positive charge with the proposed stoichiometry of the substrate of H^*+*^ = 1:2 (Nguyen et al., 1998), consequent reduction of ΔΨ may attenuate glutamate transport.

## Allosteric Regulation of the Transporter Activity

In addition to the observation that Cl^−^ ions affect the proton electrochemical gradient and in turn influence neurotransmitter uptake into SVs, Cl^−^ may directly modulate transporter activity. This concept was originally suggested from biochemical data showing that DIDS, a non-selective Cl^−^ channel blocker, inhibits Cl^−^-induced acidification and glutamate-induced acidification with different IC_50_ values (Hartinger and Jahn, 1993), leading to the hypothesis that DIDS may directly bind to VGLUT and inhibit its activity. The hypothesis could explain why glutamate uptake is low in the absence of external Cl^−^ where ΔΨ, the primary driving force for the transport, is maximal. Furthermore, ΔΨ-driven glutamate transport by reconstituted VGLUTs requires a Cl^−^ concentration of several mM, suggesting that VGLUTs are the Cl^−^-activated glutamate uniporter (Juge et al., 2010), although reconstitution of VGLUT with a proton pump suggested a contradictory conclusion (Schenck et al., 2009; Preobraschenski et al., 2014). A similar approach investigating the role of VGAT demonstrated that although GABA transport also requires Cl^−^, VGAT operates as a GABA/Cl^−^ co-transporter that is solely driven by ΔΨ (Juge et al., 2009).

## Cytosolic Factors

### Neurotransmitter Concentrations

Biochemical transport assays using SV fractions purified from native brains indicated that the transport process obeys Michaelis-Menten kinetics describing authentic enzymatic reactions (Naito and Ueda, 1985; Maycox et al., 1988). With this analogy, the velocity and extent of transport critically depends on the substrate concentrations, where greater and faster transport occurs with higher concentrations. Furthermore, like other protein-mediated transport pathways, neurotransmitter uptake into SVs is substrate saturable. In the case of glutamate uptake into isolated SVs, *K*_m_ was measured to be ~1 mM (Naito and Ueda, 1985; Maycox et al., 1988), meaning that the transport system becomes saturated with >2 mM cytoplasmic glutamate. Heterologous expression of VGLUT1-3 in non-glutamate-releasing cells confers similar, if not completely identical, affinity for glutamate uptake into isolated vesicles (Kaneko and Fujiyama, 2002; Takamori, 2006). The dependance of glutamate uptake on cytoplasmic glutamate concentrations was further confirmed in the calyx of Held synapses, where presynaptic cytoplasmic glutamate concentrations can be manipulated with a glass pipet at the presynaptic terminals. Interestingly, Ishikawa et al. (2002) loaded the presynaptic terminal with 100 mM glutamate and detected a dramatic increase in average mEPSC amplitudes by >50%, confirming that the concentration of cytoplasmic glutamate affects vesicular glutamate content. Of note, because a glutamate concentration of 100 mM may be extremely supra-physiological and may saturate the VGLUT-mediated glutamate transport, a portion of the increase in mEPSC amplitudes may be due to passive glutamate influx through VGLUT or other non-specific pathways.

From where is the neurotransmitter glutamate derived and how is it regulated? Because SVs contain large amounts of neurotransmitters, the mechanism for replenishing released transmitters at high rates of firing must be present at the presynaptic terminals. Classically, the glutamate-glutamine cycle is believed to serve as the major source of the neurotransmitter glutamate (for a review, see Chaudhry et al., 2002). In this cycle, glutamate released from the presynaptic terminals is taken up by the plasma membrane glutamate transporters that are mainly located on the surface of surrounding glial cells. Glutamate is then converted to glutamine by glutaminase. Glutamine is released by the system N transporter (SN1) and then is transported into the presynaptic terminal by the system A transporter (SAT1). Finally, glutamine is converted to glutamate by phosphate-activated glutaminase (PAG also referred as GLS1) at the presynaptic terminal. Although alterations in any step in the cycle would potentially affect the concentration of presynaptic glutamate, the rate-limiting steps have not been determined. However, despite the long-standing notion that the conversion of glutamine to glutamate is the major source of the neurotransmitter glutamate, surprisingly subtle deficits on basal glutamate transmission are seen in GLS1 knockout mice. Although the amplitude of evoked EPSCs decays more rapidly during a long train stimulation, mEPSC amplitude and duration are quite normal in GLS1-null neurons, indicating that the conversion of glutamine to glutamate in the presynaptic terminal is not an indispensable source of the neurotransmitter glutamate (Masson et al., 2006). Alternatively, α-ketoglutarate has recently been proposed as a precursor of glutamate. In fact, isolated SVs from rat brains can synthesize glutamate from α-ketoglutarate by aminotransferase in which L-aspartic acid specifically acts as an amino group donor. Therefore, enzymes involved in this pathway could potentially affect glutamate levels in the presynaptic cytosol (Takeda et al., 2012).

The synthetic pathway of GABA clearly involves the conversion of glutamic acid to GABA and is mediated by two glutamic acid decarboxylases (GAD65 and GAD67), because only trace amounts of GABA are detected in GAD65/GAD67 double knockout mouse brain (Ji et al., 1999). Consistent with this, administration of glutamate or glutamine, both of which are precursors of GABA, in cultured hippocampal neurons effectively prevents rundown of GABA transmission during recordings. Furthermore, exposure of cultured hippocampal neurons to excess concentrations of glutamine facilitates IPSCs, indicating that cytosolic GABA concentrations control vesicular GABA content (Wang et al., 2013). Recently, another non-canonical GABA synthesis pathway was discovered in midbrain dopaminergic neurons (Kim et al., 2015). This type of neuron co-releases dopamine and GABA, but GABA release does not require GABA synthesis by GAD65 and GAD67. Instead, these neurons use aldehyde dehydrogenase (ALDH1a1) to produce GABA from putrescine, which is an evolutionarily conserved pathway for the production of GABA that is also present in plants, *Xenopus*, and mammalian cells (see Kim et al., 2015). Interestingly, provided that mutations in ALDH1a1 have been linked to alcoholism in humans (Liu et al., 2011), deletion of ALDH1a1 in mice not only attenuates GABA co-release but also causes behavioral effects including increased EtOH consumption and preference of EtOH over daily water in mice, implying that diminished GABA co-release due to an insufficient GABA supply is linked to alcoholism.

### Trimeric G Proteins

Experiments using isolated SVs demonstrated that glutamate uptake is inhibited by the G-protein activator GMP-P(NH)P (Pahner et al., 2003). Kinetic experiments suggested that the inhibitor decreases both *V*_max_ and *K*_m_ of glutamate uptake. Analyses of isolated SVs derived from mice lacking Gα_o1_, Gα_o2_, Gα_q_, or Gα_11_ have demonstrated that Gα_o2_ is responsible for inhibition of glutamate uptake (Winter et al., 2005). SVs lacking Gα_o2_ exhibit normal Cl^−^-induced acidification, but have lost their Cl^−^ dependency for glutamate transport activities, indicating that Gα_o2_ may modulate allosteric activation of VGLUTs by Cl^−^. In the case of monoaminergic vesicles, the intravesicular monoamine concentration in the lumen may trigger G-protein activation (Höltje et al., 2003), providing an inhibitory feedback loop that down-regulates transporter activity when the vesicle is full. Currently, whether the same mechanism exists in glutamatergic vesicles is not clear, and if so, what types of signals, i.e., glutamate itself, protons, or other unknown factors, transfer such a signal from the lumen to the cytoplasmic side is also unknown.

### Other Factors

Besides the G proteins described above, we know little about endogenous cytoplasmic proteins that could regulate neurotransmitter uptake into SVs. Ueda and colleagues purified cytosolic proteins from bovine brains. These proteins inhibit uptake of both glutamate and GABA and were named inhibitory protein factor (IPF) αβγ (Ozkan et al., 1997). IPF appears to contain the amino acid sequence of the α subunit of the cytoskeletal protein fodrin, but fodrin itself does inhibit glutamate transport (Tamura et al., 2001). The molecular identity and physiological relevance of the function of IPFs remain to be elucidated.

## Concluding Remarks

Over the past several decades, presynaptic molecular components that regulate the quantal size have emerged. Because the amount of neurotransmitters in single vesicles may influence both spontaneous and evoked synaptic transmission, changes in quantal size may have a strong impact on brain functions including cognition, learning and memory, and behavior. Despite intensive research, we know little about the mechanisms and the rate-limiting steps underlying the formation of quanta. In particular, the mechanisms of how the vesicular neurotransmitter transporters operate and utilize the proton electrochemical gradient, as well as how Cl^−^ and other ions directly or indirectly modulate the transport activities have been enigmatic and controversial. One problem that has hampered the complete understanding of the transport systems is the lack of techniques to quantitatively manipulate the H^+^ electrochemical gradient that drives the transport. Recent technical developments, including a chimeric protein named pHoenix that allows light-driven H^+^ pumping (Rost et al., 2015) and the optimal pH probe for SV pH measurement (Egashira et al., 2015), may help to solve some of the issues discussed in this review.

## Author Contributions

ST wrote the manuscript.

## Conflict of Interest Statement

The author declares that the research was conducted in the absence of any commercial or financial relationships that could be construed as a potential conflict of interest.
